# Horizon Scan of Emerging Issues at the Intersection of National Security, Artificial Intelligence, and Human Performance Enhancement

**DOI:** 10.1007/s11948-025-00546-z

**Published:** 2025-12-03

**Authors:** Blake Hereth, Gérard de Boisboissel, Martin CM Bricknell, Maria Brincker, William Casebeer, Jovana Davidovic, Jeremy Davis, Jacob Earl, Nir Eisikovits, Daniel Feldman, Lucas França Garcia, Frédéric Gilbert, Vincent Guérin, Adam Henschke, James Hughes, Dominique Lambert, Sahar Latheef, Jonathan D. Moreno, Ian Shane Peebles, Michelle T Pham, Shira Pindyck, Ilya Rudyak, Nariyoshi Shinomiya, Neil D Shortland, Robert Sparrow, Joseph Stramondo, Laure Tabouy, Paul Tubig, David Whetham, Nicholas G. Evans

**Affiliations:** 1 Department of Medical Ethics, Humanities, & Law, Western Michigan University Homer Stryker, MD School of Medicine (USA), Kalamazoo, Michigan USA; 2 Head of the Observatory Challenges of Emerging Technologies for the Armed Forces Saint-Cyr Military Academy Research Center (France), Guer, France; 3 Center for Conflict and Health Research School of Security Studies King’s College London (United Kingdom), London, UK; 4 University of Massachusetts Boston, Boston, USA; 5 Riverside Research United States, London, UK; 6 Department of Philosophy & School of Law University of Iowa (United States) Senior Fellow, Stockdale Center for Ethical Leadership U.S. Naval Academy (USA), Annapolis, UK; 7 Department of Philosophy, University of Georgia, Lowell, USA; 8 Adjunct Lecturer, Department of Philosophy, Georgetown University, Washington, DC, USA; 9 Applied Ethics Center University of Massachusetts Boston, Boston, USA; 10 Bioethics and Human and Social Sciences Health Promotion Graduate Program, SA, Brasil; 11 Philosophy & Gender Studies Principal Leader, Ethics Lab University of Tasmania (Australia), Tasmania, Australia; 12 Catholic University of the West (UCO) and University of Angers Associate Researcher, Saint-Cyr Military Academy Research Center (France), Guer, France; 13 Assistant Professor of Philosophy University of Twente (Netherlands), Enschede, Netherlands; 14 Institutional Research, Assessment, and Planning University of Massachusetts Boston, Boston, USA; 15 Professor of Philosophy University de Namur (Belgium), Namur, Belgium; 16 ANU College of Asia and the Pacific Australian National University (Australia), Canberra, Australia; 17 David & Lyn Silfen University Professor, Department of Medical Ethics & Health Policy Perelman School of Medicine, University of Pennsylvania (USA), Philadelphia, USA; 18 School of Historical, Philosophical, and Religious Studies Arizona State University (USA), Tempe, Arizona USA; 19 Center for Ethics & the Rule of Law University of Pennsylvania (USA) , Pennsylvania, USA; 20 Center for Bioethics and Social Justice Michigan State University, Michigan, USA; 21 University of Pennsylvania, Philadelphia, US; 22 National Defense Medical College (Japan), Tokorozawa, Japan; 23 Center for Terrorism and Security Studies University of Massachusetts Lowell, Massachusetts, USA; 24 Department of Philosophy Monash University (Australia), Australian Research Council Centre of Excellence for Automated Decision Making and Society, Carlton, Australia; 25 Philosophy and the Humanities San Diego State University, San Diego, CA USA; 26 Center Gilles Gaston Granger, Université Aix-Marseille, Paris, France; 27 Department of Philosophy & Religious Studies Georgia Southern University (USA), Lowell, USA; 28 King’s Center for Military Ethics King’s College London (United Kingdom), London, UK; 29 Department of Political Science, University ofMassachusetts Lowell (USA), Lowell, USA

## Abstract

Horizon scanning is intended to identify opportunities and threats associated with technology, regulatory, and social change. Here, we report the results of a new horizon scan based on inputs of an international group of 33 participants, focusing on future issues arising from the military use of artificial intelligence (AI) for augmenting human performance. The final list of 12 issues includes topics spanning from the political (educating and training individuals to accept and work with AI), to the regulatory (issues of consent to human-AI teaming and hybridization), to security (the hackability of neural devices that connect to AI), to philosophical (the nature and phenomenology of brain-to-brain interfaces). The early identification of such issues is relevant to researchers, policymakers, military practitioners, and the wider public.

## Introduction

Artificial intelligence (AI) is expected to have profound impacts on society (Jobin et al., 2019) as applications increase across multiple areas and the power of AI becomes greater and more generalizable. In national security areas, the speed of this change and the breadth of the applications are already becoming apparent, making forecasting the impacts of AI both urgent and difficult. A key concern that arises is how AI might develop with advances in other disciplines as *converging* technological capabilities (Sententia, 2004), sometimes referred to as “integrative” (Khushf, 2006) that arise when disparate technological disciplines combine to produce novel effects. The convergence of nanotechnology, biotechnology, information technology (including AI), and cognitive science (NBIC) is significant as the capacity to understand and manipulate human biology and consciousness develops along lines that require insights from all four domains (Roco & Bainbridge, 2003). This convergence is also understood to have potential national security implications as this convergence creates novel capacities that may change the treat landscape nations must respond to (McCreight, 2013), or challenge existing governance that responds to only one of these technologies but not the others (Evans, 2019; Dando; 2020).

A perennial area of concern for converging technologies and national security is human enhancement (Roco & Bainbridge, 2003). As nations adapt to new threats and a changing world, what a warfighter looks like is expected to change as well (Hereth & Evans, 2022; Puscas, 2018). Increasingly, AI plays a role in this enhancement landscape. The increasing sophistication of brain computer interfaces is thought to include the potential for advances in drone warfare and information processing by linking warfighter brains to AI (Dinniss et al., 2018). Education and training is promised to be accelerated by using neurological feedback processed through AI to ensure that (Miranda et al., 2015). And the use of AI in exoskeleton control will enable the development of novel robotic tools that enhance a warfighter’s strength and function (Billing et al., 2021).

Despite the national security implications of the intersection of AI and human enhancement, the field remains difficult to predict and—like its progenitor disciplines (Evans, 2021; Marks, 2010)—vulnerable to hype. This makes predicting the ethical, legal, and social implications of these converging disciplines difficult to manage and vulnerable to the so-called Colingridge (1980) dilemma: when change is easy, the need for it cannot be foreseen; when the need for change is apparent, change has become expensive, difficult, and time-consuming. In recent years, methods to identify and prioritize emerging security issues arising from technology have proliferated (Boddie et al., 2015; Kemp et al., 2020; Nugraha et al., 2016; Sattler et al., 2022; Wintle et al., 2017). However, with rare exceptions such as Sattler and colleagues (2022) study of neuroscientific enhancement in the military among staff officers, very few of these explicitly much less exclusively focus on prospectively identifying ethical issues leveraging the advantages to predicting futures that come from social scientific methods.

*Horizon scanning* aims to identify upcoming opportunities and threats from technological and societal change as a means to set priorities and policy (Sutherland & Woodroof, 2009). Horizon scans frequently use Delphi panels and surveys, but more recent protocols have emerged to more accurately identify issues in convergent and emergent technical areas including conservation biology (Sutherland et al., 2006,2017), invasive species control (Ricciardi et al., 2017), poverty reduction (Pretty et al., 2010), and biological risk (Boddie et al., 2015). Horizon-scanning activities have informed funding prioritization in public policy (Kennicutt et al., 2019) and shaped the trajectory of scientific research (Kennicutt et al., 2015).

In this article we utilize the “investigate, discuss, estimate, and aggregate” (IDEA) protocol developed by Hanea et al. (2017) to identify and rank ethical issues arising at the intersection of AI and human performance enhancement in national security contexts. For the purposes of our study, we understood AI as *non-natural, simulated, algorithm-driven problem-solving technology* (Vallor & Bekey, 2017, pp. 339–340), and human performance enhancement (HPE) as *improvement of (contextualized) human performance beyond its statistical or species-typical norms* (Savulescu & Bostrom, 2011; cf. Evans et al., 2021). Participants developed a long list of issues, scored those issues anonymously based on likelihood, impact, and novelty to create a short list of 20 issues then discussed as a group. After open discussion and further deliberation, participants re-scored these issues for their priority and significance.

Here, we describe the top 12 issues identified in the AI and HPE horizon scan of 2022. To avoid a false sense of precision, issues are not ranked but rather grouped on timescales at < 5, 5–10, and > 10 years. We discuss the ethics of these cases, and opportunities for responding to the issues presented. We then discuss the limits of our study, and the potential for future engagement with the ethical issues raised herein by researchers, policymakers, and the public.

## Methods

Our study utilizes the Investigate, Discuss, Estimate, Aggregate (IDEA) protocol. In this process, participants investigate and submit candidate issues, privately and anonymously score the gathered issues, and discuss their thinking with others. They then provide a second score rhar is mathematically aggregated (Hanea et al., 2018a). The IDEA protocol advances on Delphi studies and has been widely used in emerging technological futures (e.g. Kemp et al., 2020). Aside from seeking a shared understanding of technical terms, consensus is not sought during discussion and scores are kept anonymous during both rounds. This is done to avoid undesirable group pressures from distorting individual judgements. Our protocol evolved over three phases: i) recruitment and issue gathering; ii) initial scoring; and iii) workshop preparation, deliberation, and re-scoring.

### Phase One: Recruitment and Issue Gathering

We recruited 33 participants from the United States, Japan, Australia, France, Belgium, the Netherlands, England, and Brazil. Recruitment was done via the core project team and a snowball sampling method from other experts as recruited. The panel aimed to ensure a balance across areas such as philosophy, military affairs, computer science, political science, neuroscience, and armed forces personnel. We asked participants to provide issues that were novel, plausible and high-impact, and at a specific level of granularity. Participants were asked not to focus on a general topic, such as ‘artificial general intelligence’ research, nor on multiple topics simultaneously. Instead, they were guided to focus on one area within a general topic and its implications, such as AI-human teamed decision-making in targeting operations. After duplicates were merged, a “long-list” of 62 issues was generated from the initial submissions.

### Phase Two: Scoring

Participants voted on the ‘suitability’ of these issues using a score of 0–1,000. Each score was unique (no identical scores within a given score-sheet). The suitability scores reflected a combination of plausibility, novelty and impact. Novelty was also captured by respondents noting whether they had heard of the issue previously.

Scoring was performed by all participants on the long list of issues. All anonymized scoresheets are provided in Supplementary file 1. Participants were also able to provide comments on the different issues on the voting sheet. Comments were retained by the project team for workshop discussion. We calculated the z-scores for each participant’s issues scores. Z-scores are created by subtracting the mean and dividing by the standard deviation for each issue against the participant’s set. Rather than mere averages, z-scores are sensitive to large variation in scoring so that outliers or long tails do not strongly influence scoring in a relatively small group. We then ranked the average z-scores across the issues and selected the highest ranked 12 for discussion by the group.

### Phase Three: Workshop Preparation, Deliberation, and Re-Scoring

The 12 issues with the highest scores were kept as a part of a shortlist. These were sent back to participants, who were also assigned ‘cynic’ roles for each issue. The role of cynic involves deeper background research into the topic. Each issue had at least two cynics, ensuring that at least three participants (the cynics and proposer) had an in-depth knowledge of the area—cynics were selected so that no one received their own ideas (i.e., as is proper) back. The workshop was held in Boston on June 17–18, 2022, with 20 participants who were available during the study period (Table 1).

**Table 1:** Horizon scan demographics and participation

Characteristics	Phases one and two	Phrase three (workshops)
Sample Size	33	20
Gender Balance	24 male participants (~73%) and 9 female participants (~27%).	15 male participants (75%) and 5 female participants (25%).
Geographical Coverage	8 countries (US, UK, France, Australia, Brazil, Japan, Netherlands, and Belgium).	7 countries (US, UK, France, Australia, Japan, Netherlands).
Disciplinary Distribution	26 participants from humanities and social sciences (~79%) and 7 from natural sciences (~21%).	14 participants from humanities and social sciences (70%) and 6 from natural sciences (30%).

Discussions were moderated by members of the project team. Participants were also asked to vote on whether one issue duplicated another, and whether those issues should be combined in the final shortlist.The shortlist was ultimately reduced to 12 consensus of the workshop group in response to issues that arose during deliberation. Participants were then given time to discuss the final list and whether any amendments were needed. The final list (Table 2) was approved for drafting and publication by the workshop, after the combination of both workshop’s results. The participants then anonymously ranked the issues for a second time.

**Table 2:** Identified shortlist of issues by timeframe

< 5 years	5–10 years	>10 years
AI as a tool to enhance military decision-makers	Artificial Intelligence as a selection tool in recruitment, training, and deployment^1^	BCIs and hackability
Adequate training for people working with AI assisted systems	Issues Surrounding Coercion and Consent	Sensory enhancement and the moral harms of disenhancement
BCIs, prosthetic limbs, and personal identity	The use of AI in war gaming	AI-enabled neurocognitive monitoring and neuromodulation
	Maintaining non-combatant immunity	Brain to Brain Interfaces (BBIs) or a network of connected brains; blurring the boundaries of individual responsibility
	Issues surrounding neuroenhancement opportunities pre- and post-deployment	

^1^ Some participants noted that this is already occurring in industrial and military contexts. However, the consensus was that this would become more impactful in the 5-to-10-year period.

## The Issues Most Relevant within Five Years

### Artificial Intelligence as a Tool to Enhance Military Decision-Making

AI is predicted to enhance military decision-making by undertaking ‘human-style’ tasks in a way that is quicker, more precise, and more reliable than humans. This technology can lead to automatic or delegated decisions that would be classed as ‘military,’ such as shooting a ‘military target’ (e.g., an incoming missile). The speed which these decisions might need to be made could justify ‘removing the human from the loop’ (e.g., an anti-missile system on an aircraft carrier responding to an incoming hypersonic missile) (Whetham & Payne, 2019). However, there is a risk that this AI use of HPE might remove some of the safeguards implicit in international law or other legal or ethical constraints on the use of force in conflict– so called ‘human in the loop.’ It might also allow the collation and storage of information and metadata that would allow governments to exert control over populations in a way that would bypass constitutional and democratic safeguards.

### Adequate Training for People Working with AI-Assisted Systems

Even if AI is only informing decisions made by humans, it requires that those humans understand what the AI is saying. For example, when assessing a threat, what would a ‘59% chance of attack’ mean? Is that a coin toss? Does it meet or fall short of the certainty required to employ lethal force? It is essential that those whose work is adjacent to AI systems, as well as leadership, understand the capabilities of AI (Gehlhaus et al., 2021) The ‘black box’ challenge of some forms of AI, moreover, means that the user may not be able to find out how the decision is made but, to be able to rely on it, they must have a way of determining how much trust they can place in it (Burrell, 2016).

For example, an infantryperson may not need to understand how an answer is reached by an AI advisor, but they *do* need to know how to best incorporate what the instrument is saying into the decision about what to do next. Even though a 30% prediction of something happening is not “wrong” if that thing does happen, failure to occur can still undermine trust (Department of Defense, 2022). Operators will thus need to have some understanding of biases regarding probability and value: A 30% chance of winning an election is bad odds and would lead people to change whether they can be bothered to vote, whereas a 30% chance of surviving cancer might be enough to motivate you to fight on. Rather than reducing the training and education burden, the increased use of AI assisted decision-making may require more educational investment to ensure operators understand their systems. For human performance to be enhanced using AI, it is necessary that the users have calibrated trust with respect to the system they are using. Depending on the AI they are using, what maximizes trust will vary, and thus carefully catered training is necessary for humans using or teaming up with such AI.

### BCIs, Prosthetic Limbs, and Personal Identity

Prosthetic limbs controlled through Brain Computer Interfaces (BCIs), which increasingly utilize AI for their function, introduces questions about body ownership, self-image, and self-understanding. BCI innovations may blur the boundaries between a user’s sense of identity and computerized implantable devices in unprecedented ways (Walker & Sparrow, 2023). For instance, to what extent does an implantable BCI device become incorporated into a user’s sense of self (Gilbert et al., 2019b)? To what degree are potential harms correlated with implantable BCI devices invading the user’s individual capacities (Gilbert et al., 2017)? BCI-enhancing technologies offer great control at the level of neural circuits, but the extent to which this grasp on neuronal function affects the user’s sense of control at the psychological level remains uncharted territory. There is a need to investigate how notions such as personality, identity, agency, autonomy, authenticity, and self may be iatrogenically impacted (Tabouy et al., 2023; cf. Gilbert et al., 2021).

## The Issues Most Relevant within Ten Years

### Artificial Intelligence as a Selection Tool in Recruitment, Training, and Deployment

It will be possible to combine a variety of sources of information on an individual’s genetics, health and biological markers, psychometric profile, etc., as a summary of their current and potential future health mapped against a population sample. This could have very significant implications for HPE by selecting (in/out) individuals for HPE. Armed forces are custodians of individuals’ health records during military service. They also access these records prior to military service to detect any health conditions that might medically preclude such service. These records are transferred to other custodians after military service, e.g. veterans health services. Armed forces may have other personnel records and the combination might lead to a potential for misuse as the term ‘human or personnel capability’ is increasingly being used alongside military equipment to describe military power. Additionally, the potential for AI to generate disparate impacts between groups based on how algorithms cluster variables (e.g., in the case of COMPAS and predicting recidivism) could impact how AI is used to recruit, train, and deploy (Chouldechova, 2017; Dressel & Farid, 2018).

### Issues Surrounding Coercion and Consent

Soldiers—particularly those deployed to combat environments—accept a broad range of risks throughout the course of their work. Sometimes, whether a given soldier has a heightened risk is a product of whether their comrades are prepared to aid in their defense (e.g., by providing cover when the soldier is exposed). This issue was played out over vaccine mandates within armed forces, and between them internationally. As novel enhancement technologies become more widely available, we are likely to encounter a decision point wherein leaders will have to decide whether to make these enhancements mandatory, or whether they will remain optional. Much of the discussion surrounding informed consent seems to gravitate toward the former scenario of mandated enhancement and focus on its coercive dimension (Latheef & Henschke, 2020). However, influence may not rise to the level of coercion, and we ought also to pay close attention to cases where warfighters feel immense pressure to accept enhancements. Their unit mates may exert subtle (or not-so-subtle) forms of pressure; soldiers may feel they risk their career or promotion opportunities by declining; they may be viewed as disloyal or less prepared by declining; they may be turned down for desirable assignments if they decline; and so forth (e.g., sect. Phase Two: Scoring, Phase Three: Workshop Preparation, Deliberation, and Re-Scoring, 3.5, and Issues surrounding coercion and consent).

### The Use of AI in War Gaming

War gaming has traditionally been an important strategic tool for thinking about how international crises could emerge or how they will unfold. Statistical machine learning has great promise in streamlining war gaming, potentially reducing the number of participants, length of time, and costs of such procedures. What are the advantages and disadvantages of using AI for war gaming? To the extent that statistical machine learning is essentially conservative, and to the extent that its models are based primarily on historical data and preferences, do we put ourselves at a disadvantage when we rely on AI-based war gaming (Paszkiel, 2022)?

### Maintaining Non-Combatant Immunity

The ability to distinguish between combatants and noncombatants is the key part of *jus in bello*, especially within asymmetric wars that embed combatants with civilian populations. How can artificial intelligence help distinguish permissible targets, and what kind of proxies and models will be used to identify noncombatants? Is there anything important lost in outsourcing these decisions to algorithms? Even if algorithms eventually economize on civilian deaths, one thinks of George Orwell’s famous story from the Spanish Civil War, detailing a decision not to shoot at a fascist soldier using the bathroom. Shooting that soldier would have been lawful, but Orwell did not want to shoot someone in such a vulnerable state. Will there be a place for permissible/lawful targets that might nonetheless be spared when algorithms make (or strongly recommend) decisions?

### Issues Surrounding Neuroenhancement Opportunities Pre- and Post-Deployment

Post-traumatic stress disorder (PTSD) and other combat-related psychological conditions are of great concern to both the military and broader society. Neuroenhancements could mitigate some of the most serious causes of PTSD by, for example, de-creasing a soldier’s natural empathetic response to killing, decreasing costs to civilians, etc. One potential outcome, then, is that we might witness a decline (though, to be sure, not a full elimination) in the quantity and severity of cases of PTSD and other related conditions, such as moral injury (Dobos, 2023). This possibility points heavily in favor of mandating these interventions: Not only do they generate ethically better treatment and outcomes in and around combat scenarios; they also mitigate some of the most severe psychological effects on warfighters (Giustino et al., 2016; Kolber, 2006).

However, if these interventions are optional, one wonders whether there may be fewer resources devoted to treating PTSD and other trauma-related conditions (Henschke, 2019). In other words, soldiers may be taken to have forfeited their opportunities for treatment by declining the preventive measures. Research on the issue ought therefore to be sensitive to the possibility that agencies like the VA (Veterans Affairs), as well as internal teams in garrison and elsewhere in the military community, will view certain neuroenhancements as preconditions for future treatment.

## The Issues Most Relevant in 10 Years and beyond

### BCIs, Autonomy, and Hackability

Most BCIs’ value will depend on updated or even continuously monitored data for optimal performance: these BCIs will need a connection to online data sources for these updated. Another subset of BCIs (e.g., BCIs that help one perceive via echolocation or ‘see’ in the infrared range might run of closed machine learning models built into the chip) but will nonetheless be equipped with the *option* to upload new data. In both cases, access to BCIs creates a new range of vulnerabilities for the soldiers: namely, the fact that such BCIs could be changed on the grounds of military necessity without individual consent or hacked. This use of data, and its means of update, presents novel vulnerabilities that are grand in scale, where a single hack could simultaneously cause harm to hundreds or thousands of individuals simultaneously (e.g. Evans et al., 2022).

### Sensory Enhancement and the Moral Harms of Disenhancement

AI-powered BCIs may be used to alter a soldier’s perceptual apparatus, such as giving them a new or improved sensory function. Imagine a soldier with a sensory BCI providing them with the capacity for electroreception. This is useful for sensing the presence of enemy combatants when they are not detected through our visual or auditory modalities. The gain or loss of sensory capacity, however, pervasively shapes how we perceive and interact with the world, which then pervasively shapes our self-conception. Electroception may thus become one of the embodied capacities from which (a class of) enhanced warfighters derive a meaningful sense of self. After separation, a soldier might be required to undergo disenhancement through BCI removal or deactivation, for security reasons. Although warfighters may be made aware that they will lose their BCI capacities after their service, they now facing the prospect of having a kind of disability (Evans et al., 2021) imposed on them through losing capacities that shape their experience and identity. Is compulsory disenhancement morally justified when the BCI is deeply integrated into a soldier’s meaningful sense of self and bodily integrity?

### AI-Enabled Neurocognitive Monitoring and Neuromodulation

Recent scientific advances have enables detailed assessment and modulation of neurocognitive functions. These techniques and technologies—in tandem with additional human terrain means and information (e.g., social media monitoring, biometric assessment, big data analytics, AI)—can be employed in intelligence collection to establish patterns of human neurocognitive and behavioral processes in both targeted individuals and groups. Such information could then be used to increase the precision of narrative and messaging approaches to affect adversaries’ social-cognitive processing and, by extension, their resulting decisions and actions.

Additionally, AI-enabled neurocognitive monitoring and non-invasive forms of neuromodulation (e.g., neurofeedback based transcranial electrical or magnetic stimulation) can enhance intelligence operators’ analytic and influence capabilities by optimizing signal-to-noise detection capabilities and mitigating/preventing cognitive traps set by adversaries’ cognitive space manipulation. Taken together, we term the use of these emerging techniques and technologies ‘NEURINT’– neurocognitive intelligence– as an emerging field adjacent to other forms of informational gathering, analysis (e.g.- SIGINT, COMINT, etc.), that raises novel concerns of the means by which we generate intelligence capabilities and influence.

### Brain-to-Brain Interfaces (BBIs) Blurring the Boundaries of Individual Responsibility

In 2019, research in the field of neuroscience demonstrated proof of concept for the first ever Brain-to-Brain Interface (BBI), which can connect up to 5 individuals via non-invasive BCI (Jiang et al., 2019). Proof of concept has demonstrated that BBIs can be used via the Internet and can connect individuals located in different countries (e.g., users were in France and India, see Grau et al., 2014). This technology has the protentional to revolutionize battlefield technology by enhancing collaboration, increasing situational awareness, enhancing decision-making, and providing greater insight into an individual’s cognitive states to detect cognitive bottlenecks prior to their occurrence. BBIs also bring to the forefront ethical concerns regarding how we assign responsibility for actions and outcomes that are the direct result of decisions made by two or more individuals connected to a BBI. The key point here is that *BBIs blur the boundaries of individual responsibility* (Latheef, 2022). Who ought to be held responsible for the decisions made when connected to a BBI? From this theoretical launch point, it is worthwhile to analyze whether a *collective* model of responsibility is better suited than attributions of individual responsibility.

## Discussion

### Risk

Several overarching points emerged from our discussion. First, participants consistently distinguished between AI as a decision *aid* and as a decision-*maker*. There is a broad literature on the role of machine autonomy in military settings that distinguishes between removing humans altogether (“off-the-loop”); requiring meaningful human control (“in-the-loop”); or in supervisory roles (“on-the-loop”) relations between artificially intelligent systems and human operators or teammates (Dresp-Langley, 2023). This distinction impacted several areas of ethical concerns such as wargaming (BCIs, prosthetic limbs, and personal identity), where a certain threshold of confidence in the efficacy of AI actions was seen as necessary before adoption.

At times, questions of human control highlighted tensions between HPE and AI that, to our knowledge, has been unexplored in the literature. These are instances of AI use where, at a certain stage of maturity, AI may simply be relied on in with minimal or no human input (Sparrow & Henschke, 2023). Issues such as target selection (Phase one: recruitment and issue gathering, 3.4) raise questions about when AI will no longer be used to augment, but rather replace some human capabilities that are relevant to combat. In these cases, participants noted that existing paradigms of AI as performance enhancing break down when there is no longer a human to “enhance.”

These distinctions had material effects on the discussion as the study progressed. Participants reported that during the initial vignette creation and on their arrival to the workshop a standing presumption that AI would be a decision aid and that there would always be a human ‘in the loop.’ However, over the course of the discussion some relaxed or changed their position, in cases where AI might in principle present a better decision maker, and where the absence of “meaningful human control” (van der Waa et al., 2015) did not seem to present pressing and direct moral issues—such as the use of AI for logistics. Briefly discussed was the idea that, for example in the USA, the commitment to maintaining meaningful human control is only required in terms of lethal autonomous weapons, where AI for HPE may be used in a quite broad range of settings (e.g. signals, logistics). One implication of this is the idea that what is being enhanced, even with humans, is a particular decisional or other *capability*, and that in some contexts further enhancement of that capability—all other things being equal—may require removal of a human from that use case.

### AI and Autonomy

A second, related but distinct area of discussion by concerned the autonomy of human actors in human-AI networks, and in particular what the moral and psychological impact might be of humans being governed by AI in military operations (Sparrow & Henschke, 2023). This signals not just the role of autonomy in promoting human well-being by giving individuals control over their choices as in issue, but autonomy *as a component* of well-being (e.g. Griffin, 1988).

Two other participants noted that sometimes, enhancing soldier abilities can not only augment the capabilities of the agent but also can ensure the preservation of their autonomy and agency while operating alongside AI systems. However, when considering the prospect of completely replacing critical and vital soldier capacities with AI, there a risk emerged that agents may lose their autonomy by becoming more of a passive entity within the decisional system (Miletic & Gilbert, 2020). Participants reflected on the risk of technological alienations and estrangement, epistemic dependency, and even undermined soldier self-determination, in worst case agent controlled entirely by the technology loop. A connection was made to the ways that alienation is tied to post-traumatic stress (Belew, 2018; Usry, 2019), and how replacement of solider within key features of a system could harm soldiers during and after deployment.

Autonomy as both constitutive of and promoting well-being came into play in instances where brain-computer interfaces might confer new capacities (e.g. Phase three: workshop preparation, deliberation, and re-scoring and Issues surrounding coercion and consent) or might erode boundaries of identity or control (Artificial Intelligence as a selection tool in recruitment, training, and deployment, Maintaining non-combatant immunity). Here, recent work has shown that while there are ways in which humans in-the-loop with their BCIs may enhance feelings of control, this connection between authorization and autonomy is not straightforward. At times, the ability to know and govern certain decisions may entail loss of control, such as when a BCI informs its user of an unavoidable seizure (Gilbert et al., [Bibr CR22][Bibr CR25]). Additional work in military contexts and specific use cases would be needed to examine where a sense of autonomy emerges, and role it plays for individuals: one such case might be when AI enhancement enables individuals to control large numbers of drones and focuses the deleterious moral burden of killing onto a very small number of individuals (Hereth & Evans, 2022).

### Constitution of Enhancement

Human performance enhancement was viewed quite broadly by participants. Surprisingly, conventional debates around treatment and enhancement were not discussed owing to the framing of the study, and our focus on warfighters. One participant did note that in some cases the treatment-enhancement distinction might become relevant if warfighters wounded during their service received interventions that both restored some of their functionality and gave them the tools to perform the existing or other tasks in their service. That is, some interventions might take a warfighter from below-baseline (“therapy” in some senses) but rather than merely restoring function, bring them well beyond baseline (Evans et al., 2021)—a BCI that restores ambulatory function but also enables communication with AI, for example.

In some cases, enhancement was taken to be contiguous with existing training and education methodologies (Miranda et al., 2015). But in others, such as the use of implantable BCIs or related technologies, the enhancement was considered more radical and medically invasive, but also potentially invasive to individual autonomy and privacy. Given the range of AI support tools in development by state militaries, what constitutes as performance-enhancing AI was seen to be a surprisingly broad set of algorithms and tools. Considerable clarifying discussion was engaged by the assembled group to articulate which AI applications each topic did or did not cover; how they related to HPE; and their applications in the time frames given in the scan. The use of BCIs (Phase three: workshop preparation, deliberation, and re-scoring, Artificial Intelligence as a selection tool in recruitment, training, and deployment, Issues surrounding coercion and consent), for example, can relate to HPE, but not always AI; those that do relate to AI are not always HPE.

This served to clarify, however, questions participants had around whether there were *de minimus* interventions that enhanced function beyond baseline (one sense of “enhancement”) but were noninvasive, nonimplanted, and short term; versus those that required surgical (particularly neurosurgical) interventions, had long-term effects, and were irreversible. Because of the substantial penetration of AI into all areas of warfighting (e.g. Scharre, 2018), questions around definitions focused less on classical accounts of capacity and function, and more on the degree to which an act constituted an “intervention” per se.

### Feasibility and Desirability

Further debate emerged around whether a) technologies would reach maturity sufficient for military use, and b) whether state militaries would ever *choose* to use those mature technologies. For example, in the context of potentially invasive BCIs, the prospect of long-term adverse events in healthy individuals was raised as a limiting factor where noninvasive methods might suffice. While a recent study has shown a comparable safety profile with other neurologic devices (Rubin et al., 2023), these are tests on patients with severe motor for whom the balance of risks and benefits to quality of life may be quite different to those for whom, for example, a BCI is intended for national security applications.

The distinction between AI and artificial general intelligence (AGI) was seen to partly determine the feasibility and timeframe of a vignette. The use of AI in training and recruitment (Artificial Intelligence as a tool to enhance military decision-making) or the use of BCIs impacting identity (Phase three: workshop preparation, deliberation, and re-scoring) are existing issues in limited use cases, today. However, the development of these technologies into mature technologies was determined to be some years or even decades off, and tied in part to AGI’s developmental trajectory. It was made clear further that in some cases timescale of advances in, say, computer science, was not reflective of time to conversion to applications in defense environments, which are often slower, more selective, and have different norms than civilian acquisition of new technologies.

### Cross-Cutting Issues

A recurring issue in deliberations was the status and role of enhancements post-deployment or post-service. This was raised in the context of BCIs (Phase three: workshop preparation, deliberation, and re-scoring), cognitive enhancement (3.5), and sensory enhancement and disenhancement (Issues surrounding coercion and consent). The driving concern was that enhancement for national security purposes, particularly those that interact with military AI (whether specific to the service or repurposed to use national security-apt data), may confer changes to individual’s capacities that are long term and irreversible. Determining post-service obligations to enhanced warfighters is thus a critical issue for which policy could precede technology, by clarifying the role of health and welfare agencies that service veteran populations in addressing challenges for enhanced warfighters post-service. These post service questions affect, in turn, questions of meaningful consent insofar as enhancement may affect warfighters in new and serious ways, for life. These were not explored in detail, but would be part of future comparative work between national approaches to social welfare.

Finally, while informed consent was described as a mid-term issue for HPE, concerns about informed consent—and what constituted “informed” for individuals working with complex AI—pervaded discussion. At times, thresholds for being informed reduced to understanding guidance delivered form AI as a decision maker (Phase one: recruitment and issue gathering) or partner/adversary in wargaming (BCIs, prosthetic limbs, and personal identity). At other times, deeper concerns about being versus *feeling* informed say, through a hackable BCI (Artificial Intelligence as a selection tool in recruitment, training, and deployment) or another’s thoughts through a BBI (Maintaining non-combatant immunity) influencing how we understand and identify with the information we have received, were discussed.

### Meta-Issues

A number of meta-issues were raised regarding methodology in ethical and philosophical analysis of emerging technologies. In particular, the distinction between individual responsibility and institutional (or, structural) responsibility was seen to have bearing on the topic of AI for HPE. For example, is AI better understood and evaluated using an individualist framework, a structuralist framework, or some combination (e.g. Lazar, 2023)? The individualist/structuralist distinction was important for understanding what sort of methodologies are most appropriate for evaluating the phenomena discussed in the vignettes.

This was related to ongoing methodological debates about whether social critics should prioritize moral philosophical investigation, which tends to favor analysis at the individual level and draw from the conceptual tools of moral philosophy (e.g. Garcia, 1996), or political philosophical investigation, which takes structures as the primary unit of analysis and favors the conceptual tools of political philosophy (e.g. Shelby, 2014). This exercise on AI for HPE was in a military context, which is deeply embedded in the political, including justifications for enhancing persons for the purpose of war (Hereth & Evans, 2022). However, many of the concerns raised (e.g., responsibility, autonomy, identity, and the moral harm of disenhancement) require serious evaluation that utilizes the tools of moral philosophy. The group was thus split on the degree to which our appropriate locus of analysis for a given topic should occur at the systems level, individual, or both—and if the latter, how these two levels of analysis should interact.

## Limitations

While useful, our horizon scan has limitations. The IDEA protocol is a relatively recent evolution of Delphi survey techniques, and these can over emphasize the impartiality of expert jusgement (Sackman, 1975). Our method is justified in part because of the lack of structure to the problem space, which is where IDEA has been found to improve group judgement and outperform other forecasting methods (Hanea et al., 2017) A recent review of a long-term Delphi in predicting developments in the health sector 14/18 identified issues were accurately assessed (Parente & Anderson-Parente, 2011).

Our theme, then, is well suited to the modified IDEA method used here for several reasons. There are broad definitional (e.g., what constitutes AI or human enhancement) challenges in our domain, as well as deep technical uncertainties that need resolution prior to technological maturity. The pre-test, deliberation, and post-testing method of the IDEA model is designed to select of issues based on ranked aggregation and then use structured deliberation to resolve superficial (e.g., basic definitional debates about AI) and deep (e.g., the overall plausibility of BBIs) divergence between parties (Hanea et al., 2017).

This is not to say that the methods used here are without limits. There may be significant differences among researchers in their outlooks regarding the substantial progress and future development of AI, among other enabling technologies. Therefore, the time course of near-future predictions may be inaccurate given the broad range of expertise in the group: Given the range of expertise required for this scan, it is unclear whether agreement signifies an accurate judgment.

Gender sampling may also play a role in biasing the assessments of technology, recognizing that gender diversity leads to better team research outputs (Nielsen et al., 2017). Initial sampling was conducted at gender parity and combined with snowballing arrived at approximately 40% female and nonbinary participants. However, acceptance rates for non-male participants were disproportionately lower—45% compared to 70%—resulting in a skewed final sample. Part of this may be structural features of the field in which male-identifying participants typically have more time to participate in travel: in the nonproliferation community significant attention has been devoted to characterizing the sources of the gender gap in that expert community and determines professional role can be determinative in this way (Brown & Considine, 2022). Timing may also be a factor: there is evidence showing that women suffered the burdens on research efficacy due to COVID-19 disproportionately to men (Davis et al., 2022). In any case, future IDEA studies might use alternate sampling strategies to prioritize inclusion of gender-diverse experts.

Divergence in background ethical principles may also influence the content or bring to the surface distinct aspects of the thought process in future forecasting, especially in particularly ethically salient domains such as national security and armed conflict. Different ethical commitments, for example, entail different considerations of impartiality of risk and the moral status of future persons, among other, which may lead to divergences in the estimation of risks inhering to scenarios (Robinson et al., 2021). Future work in using horizon scans in ethics might involve accounting for value commitments of each participant, for example, through survey techniques to establish divergence in value commitments between participants and provide a basis for deeper analysis in cases where the community diverge around the time scale or importance of issues.
